# Comparison between coronary plaque 64-slice spiral CT characteristics and risk factors of coronary artery disease patients in Chinese Han population and Mongolian

**DOI:** 10.12669/pjms.294.3618

**Published:** 2013

**Authors:** Zhigang Bai, Xiaoguang Yang, Xiaodong Han, Peide Dong, Aishi Liu

**Affiliations:** 1 Zhigang Bai, Department of Radiology, Affiliated Hospital of Inner Mongolia Medical University, Hohhot, 010059, P. R. China.; 2 Xiaoguang Yang, Department of Radiology, Affiliated Hospital of Inner Mongolia Medical University, Hohhot, 010059, P. R. China.; 3 Xiaodong Han, Department of Radiology, Affiliated Hospital of Inner Mongolia Medical University, Hohhot, 010059, P. R. China.; 4 Peide Dong, Department of Radiology, Affiliated Hospital of Inner Mongolia Medical University, Hohhot, 010059, P. R. China.; 5 Aishi Liu, Department of Radiology, Affiliated Hospital of Inner Mongolia Medical University, Hohhot, 010059, P. R. China.

**Keywords:** 64-slice spiral CT, Coronary artery disease, Coronary angiography, Mongolian

## Abstract

***Objective*** ***:*** To compare the coronary atherosclerotic plaque 64-slice spiral CT characteristics and the risk factors of Han (in Inner Mongolia) and Mongolian coronary artery disease patients.

***Metho*** ***d*** ***s:*** The plaques of 126 Mongolian and 269 Han patients were analyzed by 64-slice spiral CT coronary angiography. Their gender, age, height, body mass, the history of hypertension, diabetes, smoking and family diseases, the levels of triglycerides (TG), total cholesterol (TC), high density lipoprotein cholesterol (HDL-C) and low density lipoprotein cholesterol (LDL-C) were compared.

***Results:*** The incidence of plaques (P <0.05), the proportion of plaques in the circumflex branch (P <0.05), the proportion of medium-severe lumen stenosis induced by plaques (P <0.05), and the proportion of obstructive plaque involved multi-branch (P <0.05) of the Mongolian patients were higher. The plaque compositions of the two groups did not differ significantly (P> 0.05). The body mass index of the Mongolian patients was higher (P <0.05). The hypertension, diabetes, smoking history, TG, TC, HDL-C and LDL-C of the two groups did not differ significantly (P> 0.05).

***Conclusion:*** The higher incidence of coronary atherosclerotic plaques and the more severe lesions of the Mongolian patients may be related to their higher body mass index.

## INTRODUCTION

Social and economic development, improved living conditions and population ageing are the hotbed of coronary artery diseases.^1^ Catheter angiography has been traditionally utilized to compare the coronary artery lesions between different nations.^2^^-^^4^

In this paper, the coronary atherosclerotic plaque characteristics and the risk factors of coronary artery disease patients in Chinese Han (in Inner Mongolia) and Mongolian were compared by 64-slice spiral CT. The possible reasons accounting for coronary artery diseases were also explored, which will provide reference for the prevention of coronary artery diseases in different nations.

## METHODS

***General Information: ***126 Mongolian patients (M: 82; F: 44) aging 35-80 (58.32±9.66) who had been diagnosed as coronary artery diseases by 64-slice spiral CT in our hospital from January 2010 to December 2011 were selected. Another 269 Han patients aging 33-81 (59.94±9.07) with the same sex ratio (M: 180; F: 89) who had been diagnosed as coronary artery diseases by 64-slice spiral CT were also included. The patients with poor-quality CT images and those after coronary stent implantation or bypass graft surgeries were excluded. All the subjects were informed of the tests.

***Test methods:*** The patients underwent 64-slice spiral CT coronary angiography. Retrospective ECG gating was used for enhanced scan. 75-100 mL of nonionic contrast agent iopamidol (Bracco SPA, Zhunzi J20030122, 100ml: 30g (iodine)) was bolus-injected into the antecubital vein at the flow rate of 4.0-5.0 mL/s by a high-pressure syringe, which was followed by the injection of 30 mL saline at the same rate. The delayed scan time was determined by automatic triggering with the thickness of 0.625 mm and the scanning speed of 0.35 rps. The raw data were imported into ADW4.2 workstation. The best the ECG window was selected to perform volume rendering (VR), multiplanar reformation (MPR) and maximum intensity projection (MIP) reformation. Plaque sites, types, and the stenosis degrees of involved vessels were independently analyzed by two radiologists unaware of the clinical data. The inconsistent diagnoses were checked and corrected.^5^

***Evaluation method for atherosclerotic plaques:*** Coronary artery atherosclerotic plaques were analyzed by the modified segmentation method of American Heart Association.^6^ Thirteen major segments of the coronary artery tree were evaluated. The involved vascular segments of right coronary artery (RCA), left main artery (LMA), left anterior descending branch (LAD) and left circumflex branch (LCX) were counted. Atherosclerotic plaque is defined as a structure which is independently visible on at least two planes and is distributed in the coronary artery wall that can be separated from the coronary artery lumen and the surrounding tissues. The plaques were classified into the following types according to CT values: ^7^ soft plaque: CT value <60 Hu; fibrous plaque: 61< CT value< 119 Hu; calcified plaque: CT value ≥ 120 Hu; mixed plaque: contain at least 2 of the above components simultaneously.

The stenosis degree was estimated by the visual inspection of the narrowest vessel segment. The luminal stenosis degrees include: mild stenosis ≤ 50%, 50%< moderate stenosis<75%, severe stenosis> 75%. The plaques with the luminal stenosis ≥ 50% were regarded as the obstructive plaques and the number their involved major branches were counted.

***Coronary artery disease-related risk factors: ***Gender, age, height, body mass, the history of hypertension, diabetes, smoking and family diseases of the two patient groups were collected by questionnaires. The levels of TG, TC, HDL-C and LDL-C were determined utilizing the fasting venous bloods. Body mass index (BMI) = body mass (kg)/height (m). The patients who had been diagnosed and were receiving treatment were classified as the history of hypertension and diabetes. The history of smoking was regulated according to the standard of WHO (positive: one cigarette/d, continuous smoking> 1 y, long-term smoker ceasing smoking <6 months. Family history was regarded as positive in the case of coronary heart diseases in first-degree relatives.^8^

***Statistical analysis: ***SPSS16.0 was used for statistical analysis, the measurement data were expressed as mean ± standard deviation (), the count data were analyzed by the chi-square test, the groups were compared using t-test, P <0.05 was considered as significantly different statistically.

## RESULTS

***Comparison of the incidences of atherosclerotic plaques between the two groups: ***In the 126 cases of Mongolian coronary heart disease patients, a total of 412 vessel segments were involved, the incidence of atherosclerotic plaques was 25.15% (412/(126 × 13) × 100%). In the 269 cases of Han coronary heart disease patients, a total of 683 vessel segments were involved, the incidence of atherosclerotic plaques was 19.53% (683/( 269×13) × 100%). The incidence of Mongolian patients was higher than that of Han patients (χ^2^=8.167，P= 0.003).

**Fig.1:**
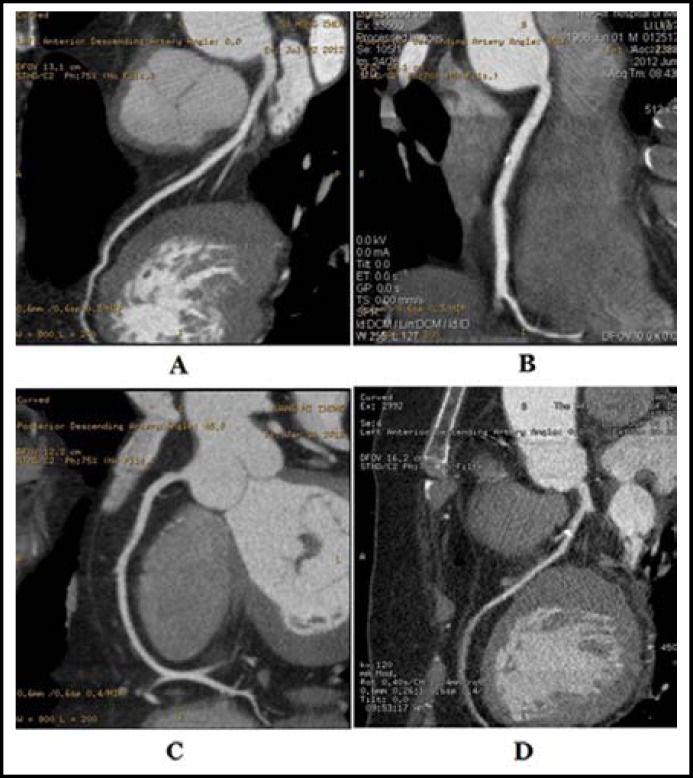
64-slice spiral CT coronary angiography images of the 4 plaque types. A: soft plaque of LMA; B: mixed plaque of middle RCA; C: fibrous plaque of proximal and middle RCA; D: calcified plaque of proximal LAD

***Comparison of the compositions of atherosclerotic plaques between the two groups:*** The types of atherosclerotic plaques found in the two patient groups were sequenced into the descending order: calcified plaque> mixed plaque> fibrous plaque> soft plaque. The ratios of the four types of plaques in the two groups did not differ significantly (χ^2^= 2.705, P =0.102) (Table-I). 64-slice spiral CT coronary angiography images of the 4 plaque types are shown in Fig.1.

**Fig.2:**
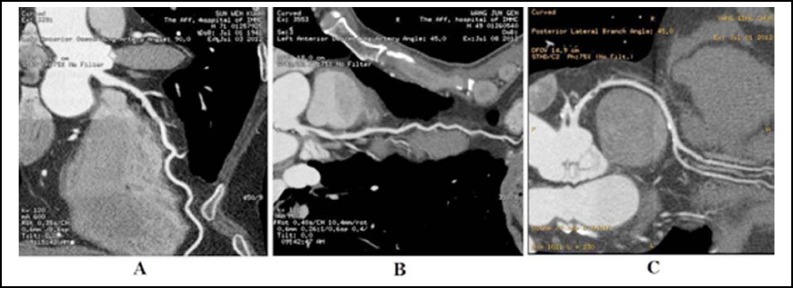
64-slice spiral CT images of luminal stenosis induced by plaques. A: mild stenosis of middle LAD; B: moderate stenosis of middle LAD; C: severe stenosis of proximal and middle RCA

**Table-I:** Comparison of the compositions of atherosclerotic plaques between the two groups

*Group*	*Soft plaque*		*Fibrous plaque*		*Calcified plaque:*		*Mixed plaque*		*Total plaque number*
	*segment number*	*ratio/%*	*segment number*	*ratio/%*	*segment number*	*ratio/%*	*segment number*	*ratio/%*	
Mongolian	24	5.83	95	23.06	116	28.16	177	42.96	412
Han	30	4.39	125	18.3	270	39.53	258	37.77	683

**Table-II:** Comparison of the sites of atherosclerotic plaques between the two groups

*Group*	*RCA*		*LMA*		*LAD*		*LCX*	
	*segment number*	*ratio/%*	*segment number*	*ratio/%*	*segment number*	*ratio/%*	*segment number*	*ratio/%*
Mongolian	123	29.85	30	7.28	150	36.41	109	26.46
Han	191	27.96	49	7.17	314	45.97	129	18.89
χ^2^	2.256		0.192		10.945		4.901	
P value	0.128		0.663		0.001		0.024	

**Table-III:** Comparison of the lesion degrees (%) of atherosclerotic plaques between the two groups

*Group*	*Luminal stenosis induced by atherosclerotic plaque*			*Number of branches involved by obstructive plaque*		
	*mild*	*medium*	*severe*	*single branch*	*double branch*	*multi-branch*
Mongolian	119 (28.88)	140 (33.98)	153 (37.14)	59 (46.83)	39 (30.95)	28 (22.22)
Han	307 (44.95)	198 (28.99)	178 (26.06)	156 (57.99)	48 (17.84)	65 (24.16)
χ^2^	12.046	5.228	20.312	8.385	0.127	9.017
P value	0	0.021	0.644	0.003	0.697	0.003

***Comparison of the sites of atherosclerotic plaques between the two groups: ***The major branches that were involved in atherosclerotic plaques in the two groups both followed the sequence: LAD> RCA> LCX> LMA. However, the plaques in the two groups were distributed differently. The ratios of plaques distributed in LCX and LAD in Mongolian patients were higher and lower than those in Han patients, respectively (Table-II).

***Comparison of the lesion degrees of atherosclerotic plaques between the two groups: ***More Mongolian patients were subject to medium and severe luminal stenosis induced by atherosclerotic plaques compared to Han patients (Table-III), and the corresponding 64-slice spiral CT coronary angiography images are shown in Fig.2. More multi-branches of Mongolian patients were involved owing to arterial obstructive plaques (stenosis ≥ 50%) compared to Han patients, whereas more single branches of Han patients were involved owing to obstructive plaques compared to Han patients (Table-III, Fig.2).

***Comparison of the risk factors of coronary artery disease between the two groups: ***The history of hypertension, diabetes and smoking, as well as the levels of TG, TC, HDL-C and LDL-C of the two patient groups did not differ significantly (P>0.05). BMI values of Mongolian patients were higher than those of Han patients (P<0.05) (Table-IV).

## DISCUSSION

Although coronary angiography is the gold standard for coronary artery disease, this expensive procedure offers severe complications. Only 30% of the patients receiving coronary angiography are in need of simultaneous interventional therapy.^9^ Coronary angiography is deficient in evaluating coronary artery disease because only the luminal contour is filled with contrast agents that indirectly reflect the lesions of atherosclerotic plaques on the artery wall.^10^ Although intravascular ultrasound can quantitatively measure atherosclerotic plaques and evaluate plaque compositions as well, it is invasive, time-consuming and expensive. In contrast, 64-slice spiral CT coronary imaging is noninvasive, sensitive and specific.^11^^,^^12^ It provides plaque location and morphology while examining arteriostenosis, and doctors can determine the composition of plaques according to the density. It has now become a new method in diagnosing coronary artery atherosclerotic plaques and predicting risks.^13^^,^^14^

The types of atherosclerotic plaques found in the two patient groups were sequenced as calcified plaque> mixed plaque> fibrous plaque> soft plaque. Besides, the plaque composition ratios of the two groups did not differ significantly. Maffei et al found that^15^ plaques containing different-density tissues could be distinguished by 64-slice spiral CT, but the measurements were affected by the partial volume effect. Meanwhile, 64-slice spiral CT coronary angiography only functions in speculating the main composition of arterial plaques, plaque lipid and fibrous cap cannot be further discerned. The results herein show that the coronary plaque compositions of the two patient groups did not differ significantly. The number of plaques found in the two groups both followed the sequence of LAD> RCA> LCX> LMA, but the ratios of plaques distributed in LCX in Mongolian patients were higher than those in Han patients. The results are inconsistent with those of Ranity et al^16^, which may be attributed to the different coronary angiography methods and inclusion criteria.

Our study shows that Mongolian patients were more prone to atherosclerotic plaques compared to Han patients. In addition, Mongolian patients were also subject to severe luminal stenosis induced by coronary plaques and more main branches involved by obstructive plaques. Yao et al^17^ reported that Han patients were subject to three branch lesion, type C lesion and total coronary occlusion.

Coronary heart disease results from a diversity of genetic and environmental risk factors, including age, gender, the family history of smoking, hypertension, diabetes and coronary heart disease, family history, blood lipid disorders and etc.^18^ In our study, the history of hypertension, diabetes and smoking, as well as the levels of TG, TC, HDL-C and LDL-C of the two patient groups did not differ significantly, but the BMI values of Mongolian patients were higher than those of Han patients, indicating that Mongolian coronary heart disease patients were seriously overweight and obese. Moreover, Mongolians prefer the diet high in animal fat and salt intake, and the two nations have inherently different backgrounds, cultures and habits as well. Similarly, the higher incidence and the more severe lesion of atherosclerotic plaques found in Mongolian patients are also associated with overweight and obesity.

In summary, traditional risk factors concerning coronary artery disease were investigated, suggesting the atherosclerotic plaque characteristics of Mongolian and Chinese Han population patients differed. Nevertheless, whether the differences are related to race and gene are still in need of further study.
